# Maternal plasma proteome profiling of biomarkers and pathogenic mechanisms of early-onset and late-onset preeclampsia

**DOI:** 10.1038/s41598-022-20658-x

**Published:** 2022-11-09

**Authors:** Hao Chen, Ingrid Aneman, Valentina Nikolic, Natasa Karadzov Orlic, Zeljko Mikovic, Milan Stefanovic, Zoran Cakic, Hristina Jovanovic, Stephanie E. L. Town, Matthew P. Padula, Lana McClements

**Affiliations:** 1 School of Life Sciences, Faculty of Science, University of Technology Sydney, Ultimo, NSW Australia; 2 Department of Pharmacology and Toxicology, Faculty of Medicine, University of Nis, Nis, Serbia; 3 Department of Gynaecology and Obstetrics, Narodni Front, Belgrade, Serbia; 4 Faculty of Medicine, University of Belgrade, Belgrade, Serbia; 5 Department of Gynaecology and Obstetrics, Faculty of Medicine, University of Nis, Nis, Serbia; 6 Gynaecology and Obstetrics Clinic, Clinical Centre Nis, Nis, Serbia; 7 Department of Gynaecology and Obstetrics, General Hospital of Leskovac, Leskovac, Serbia; 8 Institute for Biomedical Materials and Devices, Faculty of Science, University of Technology Sydney, Ultimo, NSW Australia

**Keywords:** Biochemistry, Computational biology and bioinformatics

## Abstract

Preeclampsia is still the leading cause of morbidity and mortality in pregnancy without a cure. There are two phenotypes of preeclampsia, early-onset (EOPE) and late-onset (LOPE) with poorly defined pathogenic differences. This study aimed to facilitate better understanding of the mechanisms of pathophysiology of EOPE and LOPE, and identify specific biomarkers or therapeutic targets. In this study, we conducted an untargeted, label-free quantitative proteomic analyses of plasma samples from pregnant women with EOPE (n = 17) and LOPE (n = 11), and age, BMI-matched normotensive controls (n = 18). Targeted proteomics approach was also employed to validate a subset of proteins (n = 17). In total, there were 26 and 20 differentially abundant proteins between EOPE or LOPE, and normotensive controls, respectively. A series of angiogenic and inflammatory proteins, including insulin-like growth factor-binding protein 4 (IGFBP4; EOPE: FDR = 0.0030 and LOPE: FDR = 0.00396) and inter-alpha-trypsin inhibitor heavy chain H2-4 (ITIH2-4), were significantly altered in abundance in both phenotypes. Through validation we confirmed that ITIH2 was perturbed only in LOPE (*p* = 0.005) whereas ITIH3 and ITIH4 were perturbed in both phenotypes (*p* < 0.05). Overall, lipid metabolism/transport proteins associated with atherosclerosis were highly abundant in LOPE, however, ECM proteins had a more pronounced role in EOPE. The complement cascade and binding and uptake of ligands by scavenger receptors, pathways, were associated with both EOPE and LOPE.

## Introduction

Hypertensive disorders in pregnancy are the leading cause of morbidity and mortality, affecting up to 10% of all^1^ pregnancies and include chronic hypertension, gestational hypertension, eclampsia and pre-eclampsia; all of which increase the risk of complications in both mothers and babies during gestation. Preeclampsia, in particular, is characterised by the new-onset of gestational hypertension in the presence of proteinuria or other organ damage often involving kidneys or liver. It affects 5–7% of pregnancies and causes approximately 76,000 maternal deaths and 500,000 foetal deaths^2^.

The classification of preeclampsia has been evolving over the last decade. In 2013, the American College of Obstetricians and Gynaecologists (ACOG) and International Society for the Study of Hypertension in Pregnancy (ISSHP) incorporated other symptoms/features including liver dysfunction, thrombocytopenia, cerebrovascular events or foetal growth restriction (FGR), in the absence of proteinuria, to diagnose preeclampsia^1–3^. Preeclampsia is a multifactorial and heterogeneous disorder, stratified depending on the time of onset into: (i) early-onset preeclampsia (EOPE) manifested before 34 weeks of gestation, and (ii) late-onset preeclampsia (LOPE) manifested from 34 weeks of gestation. Although EOPE and LOPE share the same clinical features, these two phenotypes of preeclampsia lead to different outcomes. EOPE is commonly associated with foetal growth restriction (FGR) and abnormal uterine artery Doppler, often leading to preterm birth and a higher risk of post-pregnancy morbidities^4,5^. On the other hand, LOPE appears to be a less severe disorder, often displaying a normal or slightly increased uterine resistance index and a lower rate of FGR^5,6^. There is no distinct delineation between EOPE and LOPE, with most patients with preeclampsia presenting elements of both pathologies, proposing a clinical spectrum.

The lack of untargeted discovery studies involving multiomics analyses impeded understanding of the molecular differences between these two phenotypes of preeclampsia. In a systematic review summarising quantitative proteomics-based studies using human samples from women affected by preeclampsia, the vast majority of studies included samples from one of the preeclampsia phenotypes only or grouped both phenotypes together, lacking comparison between EOPE and LOPE^7^. In this study, we conducted an unbiased, label-free quantitative proteomics analysis using non-depleted plasma samples collected from patients with EOPE (n = 17) and LOPE (n = 11), compared with age- and BMI-matched normotensive controls (n = 18). We also validated a subset of 17 proteins, using targeted proteomics of plasma samples from a similar groups of patients. The use of plasma samples should better reflect the pathophysiology of EOPE and LOPE as systemic conditions affected by widespread endothelial dysfunction^8^, and identify potential therapeutic targets for future personalised treatment development.

## Results

### Patient characteristics

The clinical characteristics of the participants used for untargeted and targeted proteomics are presented in Tables 1 and 2, respectively. The distribution of age and BMI were similar across EOPE, LOPE and healthy pregnancy groups, whereas gestational age (GA) at delivery was significantly lower in EOPE (31.3 ± 2.5 weeks) and LOPE (37.0 ± 1.9 weeks), compared to the healthy pregnancy controls (39.4 ± 0.9 weeks, *p* < 0.05; Table 1), in line with frequent early delivery of the baby in EOPE compared to LOPE or healthy controls^5^. Maternal blood pressure was higher in EOPE (systolic blood pressure (SBP):156.4 ± 26.5 or diastolic BP (DBP):103.1±10.7) and LOPE (SBP:144.4 ± 16.8 or DBP:96.9±11.6) compared to healthy pregnancies (SBP113.9 ± 8.5 or DBP:72.2±8.1), (*p* < 0.05). Heart rate was increased in women with EOPE (94.6 ± 29.8) compared to healthy controls (74.4 ± 6.3, *p* < 0.05; Table 1). Differences were similar in the validation groups (except there was no difference in the heart rate between EOPE and healthy control, *p* = 0.05), which included the same healthy controls (n = 18) and slightly different EOPE (n = 14) and LOPE (n = 14), groups (Table 2).

**Table 1:** Summary of patient characteristic (discovery/untargeted proteomics set).

Characteristics	EOPE (n = 17)	LOPE (n = 11)	Healthy pregnancy (n = 18)
Age (y)	34.0 ± 7.1	33.0 ± 4.0	31.1 ± 3.8
BMI (kg/m^2^)	27.6 ± 4.8	28.3 ± 3.4	24.5 ± 5.5
GA at delivery (wk)	31.3 ± 2.5 ^¶&^	37.0 ± 1.9 ^§&^	39.4 ± 0.9 ^¶§^
Number of pregnancies (no.)	1.6 ± 0.9	2 ± 0.9	1.7 ± 0.8
Systolic blood pressure (mm Hg)	156.4 ± 26.5 ^¶^	144.4 ± 16.8 ^§^	113.9 ± 8.5 ^¶§^
Diastolic blood pressure (mm Hg)	103.1 ± 10.7 ^¶^	96.9 ± 11.6 ^§^	72.2 ± 8.1 ^¶§^
Heart rate (bpm)	94.6 ± 29.8 ^¶^	82.8 ± 16.5	74.4 ± 6.3 ^¶^
**Medications**			
Methyldopa (no. [%])	17 [100%]	11 [100%]	0 [0%]
Amlodipine (no. [%])	5 [29.4%]	2 [18.2%]	0 [0%]
Dexamethasone (no. [%])	11 [64.7%]	1 [9.1%]	0 [0%]
Nadroparin calcium (no. [%])	3 [17.6%]	1 [9.1%]	0 [0%]
Diazepam (no. [%])	3 [17.6%]	8 [72.7%]	0 [0%]
Magnesium sulphate (no. [%])	4 [23.5%]	1 [9.1%]	0 [0%]
Nifedipine (no. [%])	0 [0%]	1 [9.1%]	0 [0%]
Low-sodium diet (no. [%])	11 [64.7%]	8 [72.7%]	0 [0%]

BMI, body mass index; bpm, beats per minute; EOPE, early-onset pre-eclampsia; GA, gestational age; LOPE, late-onset pre-eclampsia.

^¶^, *P* < 0.05 of a characteristic between EOPE and healthy pregnancy groups.

^§^, *P* < 0.05 of a characteristic between LOPE and healthy pregnancy groups.

^&^, *P* < 0.05 of a characteristic between EOPE and LOPE.

**Table 2:** Summary of patients’ characteristics (validation/targeted proteomics set).

Characteristics	EOPE (n = 14)	LOPE (n = 14)	Healthy pregnancy (n = 18)
Age (y)	33.2 ± 7.6	32.6 ± 3.7	31.1 ± 3.8
BMI (kg/m^2^)	28.0 ± 5.0	27.5 ± 3.7	24.5 ± 5.5
GA at delivery (wk)	31.9 ± 2.1^¶&^	36.8 ± 1.9 ^§&^	39.4 ± 0.9^¶§^
Number of pregnancies (no.)	1.8 ± 0.9	2 ± 0.9	1.7 ± 0.7
Systolic blood pressure (mm Hg)	157 ± 28.9^¶^	142 ± 15.7^§^	113.9 ± 8.5^¶§^
Diastolic blood pressure (mm Hg)	103 ± 10.9^¶^	93.5 ± 12.5^§^	72.2 ± 8.1^¶§^
Heart rate (bpm)	86.1 ± 17.6	84.6 ± 15.7	74.4 ± 6.3
**Medications**			
Methyldopa (no. [%])	14 [100%]	13 [92%]	0 [0%]
Amlodipine (no. [%])	5 [35.7%]	2 [14.3%]	0 [0%]
Dexamethasone (no. [%])	9 [64.3%]	1 [7.1%]	0 [0%]
Nadroparin calcium (no. [%])	3 [21.4%]	2 [14.3%]	0 [0%]
Diazepam (no. [%])	3 [21.4%]	10 [71.4%]	0 [0%]
Magnesium sulphate (no. [%])	3 [21.4%]	1 [9.1%]	0 [0%]
Nifedipine (no. [%])	1 [7.1%]	1 [7.1%]	0 [0%]
Low-sodium diet (no. [%])	9 [64.%]	8 [57.1%]	0 [0%]

BMI, body mass index; bpm, beats per minute; EOPE, early-onset pre-eclampsia; GA, gestational age; LOPE, late-onset pre-eclampsia.

^¶^, *P* < 0.05 of a characteristic between EOPE and healthy pregnancy groups.

^§^, *P* < 0.05 of a characteristic between LOPE and healthy pregnancy groups.

^&^, *P* < 0.05 of a characteristic between EOPE and LOPE.

### Differentially abundant plasma proteins can differentiate between the different phenotypes of preeclampsia and healthy controls

Label-free proteomic analysis of non-depleted plasma samples was conducted by measuring the relative abundance of tryptic peptides using data-dependent acquisition (DDA) mass spectrometry. The generated outputs contained 370 proteins detected across all samples with minimal percentage (< 15%) of missing values (Supplementary data 1).

**Figure 1:**
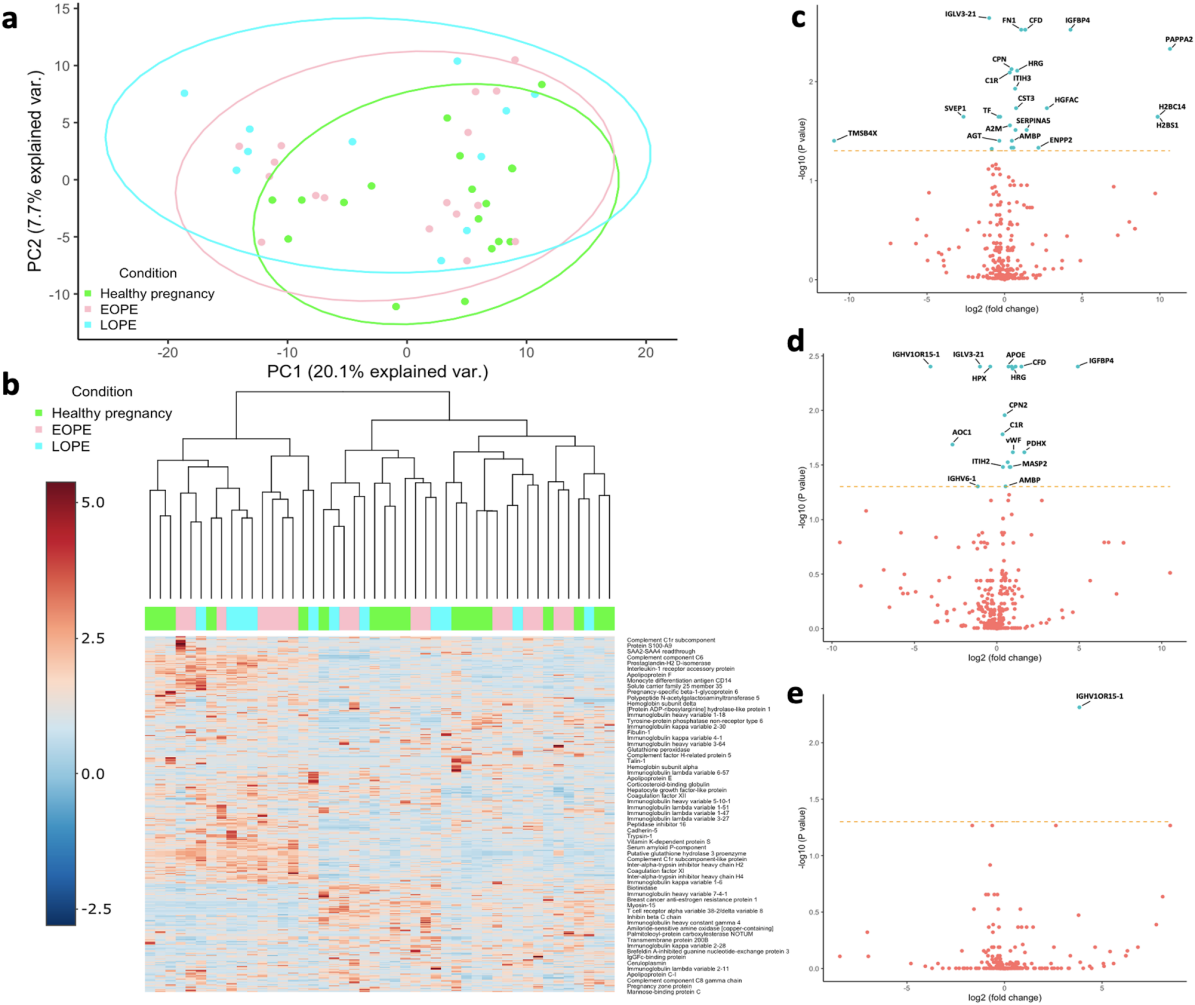
Overviews of differential expression analysis. (**a**) Principal component analysis (PCA) plot of proteomic data in EOPE, LOPE and healthy pregnancy groups. (**b**) Multigroup heatmap with hierarchical clustering dendrogram of proteomic data levels across EOPE, LOPE and healthy pregnancy groups. Volcano plots of proteomic data in (**c**) EOPE versus healthy pregnancy, (**d**) LOPE versus healthy pregnancy, and **e** EOPE versus LOPE. The differential expression (DE) analysis was performed by fitting a linear regression model adjusted for GA at delivery. DE proteins were defined as Benjamini–Hochberg adjusted *P* value < 0.05, as indicated by the proteins above the cut-off lines. Source data are provided as a Source Data file.

**Figure 2:**
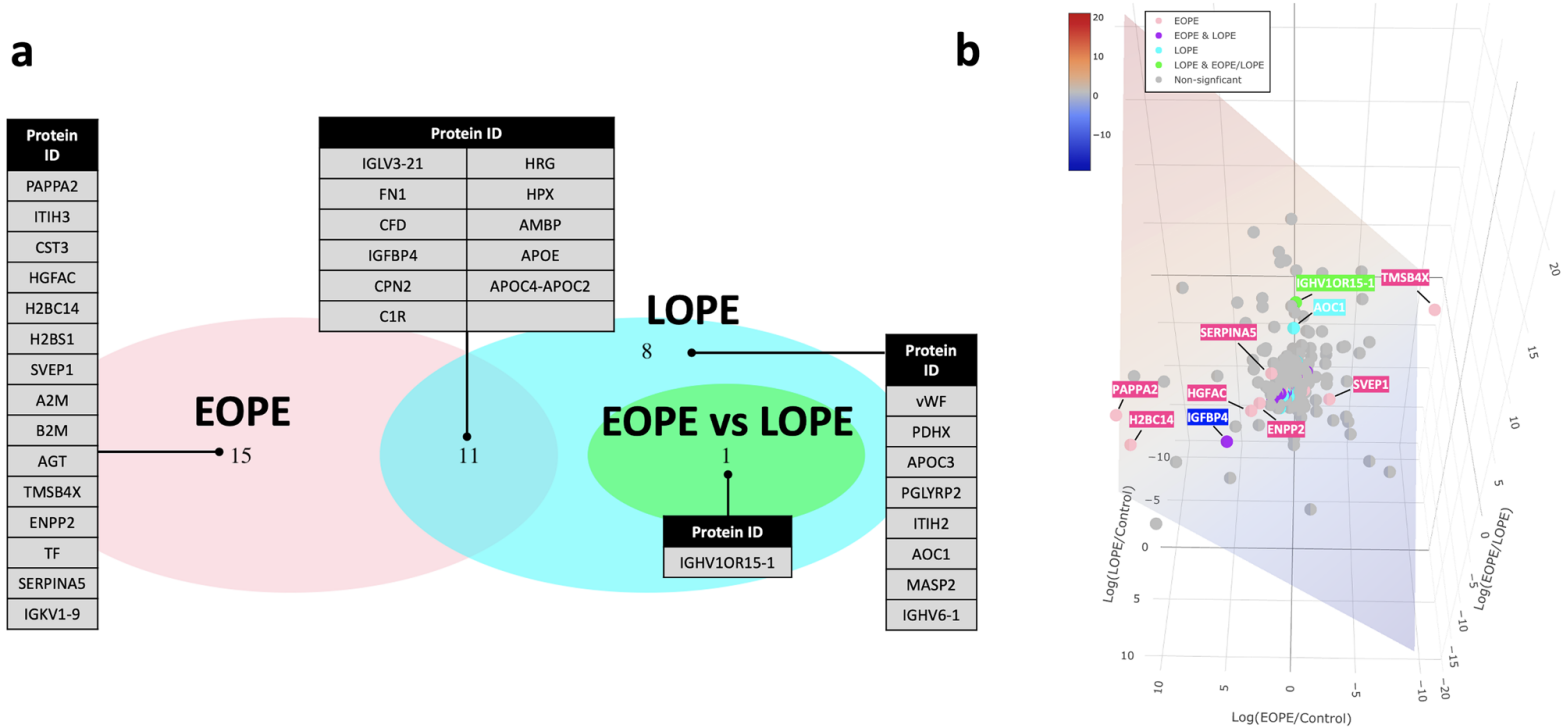
Highlighted proteins in differential expression analysis. (**a**) Triple Venn diagram summarising the differentially expressed proteins unique and overlapped between EOPE versus healthy pregnancy, LOPE versus healthy pregnancy and EOPE versus LOPE. (**b**) Three-dimensional plot of fold changes (log_2_-transformed) of all identified proteins, with regression plane filled with colour indicator of log_2_-fold changes. Source data are provided as a Source Data file.

Initially, the clustering of groups was assessed through examination of the principal component analysis (PCA) plot (Fig. 1a) and heatmap showing the unsupervised hierarchical clustering of the individual samples (Fig. 1b). Both PCA and heatmap revealed that the proteomes are heterogeneous across all the samples. Following PCA, the differential expression (DE) analysis was performed by three separate comparisons; (i) EOPE versus healthy pregnancy, (ii) LOPE versus healthy pregnancy and (iii) EOPE versus LOPE, while adjusting for GA at delivery. DE proteins were defined as FDR < 0.05. In total, there were 26 and 20 differentially abundant proteins in EOPE versus healthy pregnancy and LOPE versus healthy pregnancy, respectively, and one protein was differentially abundant between EOPE and LOPE (Table 3; Figs. 1c–e and 2a; Supplementary data 2).

**Table 3:** DE plasma proteins of preeclampsia.

Protein	logFC	FDR	Validation results logFC (*p* value)
**EOPE versus healthy pregnancy**			
IGLV3-21	− 0.9940833	0.002	− 1.10703 (*p* = 0.02)
FN1	1.07026906	0.003	0.749238 (*p* = 0.007)
CFD	1.33165459	0.003	n/a
IGFBP4	4.2479972	0.003	n/a
PAPPA2	10.6575471	0.005	n/a
CPN2	0.44690077	0.007	0.182445 (*p* = 0.008)
HRG	0.81057059	0.008	0.35572 (*p* = 0.007)
C1R	0.33304499	0.008	n/s
ITIH3	0.67823875	0.012	0.429546 (*p* = 0.02)
CST3	0.73940906	0.018	n/a
HGFA	2.72402884	0.018	n/s
HPX/HEMO	− 0.2855547	0.023	− 0.18325 (*p* = 0.007)
H2BC14	9.86191588	0.023	n/a
H2BC12L	9.86191588	0.023	n/a
TF	− 0.3914363	0.023	− 0.3227 (*p* = 0.001)
SVEP1	− 2.648795	0.023	n/a
A2M	0.34482872	0.028	n/a
SERPINA5	1.41940662	0.031	n/a
B2M	0.70236879	0.031	n/a
AGT	− 0.3379023	0.04	n/a
AMBP	0.46608993	0.04	n/a
TMSB4X	− 10.989325	0.04	n/a
ENPP2	2.17783194	0.046	n/a
APOE	0.44371805	0.046	n/s
APOC4-APOC2	0.56440844	0.046	n/s
IGKV1-9	− 0.8285979	0.048	n/a
IGHV1OR15-1		n/s	− 0.38218 (*p* = 0.004)
ITIH4		n/s	0.294583 (*p* = 0.02)
**LOPE versus healthy pregnancy**			
IGLV3-21	− 1.0237716	0.004	− 2.207995088 (*p* < 0.0001)
IGHV1OR15-1	− 4.0259103	0.004	− 0.24114122 (*p* = 0.02)
CFD	1.49090423	0.004	n/a
FN1	1.13370152	0.004	n/s
HPX/HEMO	− 0.4013146	0.004	− 0.134143098 (*p* = 0.02)
IGFBP4	4.91273421	0.004	n/a
APOC4-APOC2	0.89848759	0.004	0.285337364 (*p* = 0.02)
APOE	0.70579726	0.004	0.484743909 (*p* = 0.003)
HRG	0.95744956	0.004	− 0.315575546 (*p* = 0.005)
CPN2	0.47425523	0.011	0.317003488 (*p* < 0.0001)
C1R	0.34745024	0.017	0.475339172 (*p* = 0.002)
AOC1	− 2.6911841	0.021	n/a
VWF	0.9703592	0.024	n/s
PDHX	1.66742853	0.024	n/a
APOC3	0.66060763	0.03	0.937780283 (*p* < 0.0001)
MASP2	0.82804763	0.03	n/s
AMBP	0.52819589	0.0498	n/a
IGHV6-1	− 1.1543323	0.0498	n/a
TF			− 0.385595866 (*p* < 0.0001)
amiD	0.77746779	0.03	n/a
ITIH2	0.37228745	0.03	0.290554799 (*p* = 0.005)
ITIH3		n/s	0.434991009 (*p* = 0.008)
ITIH4		n/s	0.349781944 (*p* = 0.005)
**EOPE versus LOPE**			
IGHV1OR15-1	3.83674304	0.005	n/s
APOC3		n/s	− 0.93954084 (*p* < 0.0001)
HRG		n/s	0.671295765 (*p* < 0.0001)

n/a—not available.

n/s—non-significant.

*AGT,* angiotensinogen*; AMBP,* protein AMBP*;* *amiD,* N-acetylmuramoyl-L-alanine amidase*; AOC1,* amiloride-sensitive amine oxidase [copper-containing]*; APOC3,* apolipoprotein C-III*; APOC4-APOC2, *APOC4-APOC2 readthrough (NMD candidate)*; APOE,* apolipoprotein E*; A2M,* alpha-2-macroglobulin*; B2M,* beta-2-microglobulin*; CFD,* complement factor D*; CPN2,* carboxypeptidase N subunit 2*; CST3,* cystatin-C*; C1R,* complement C1r subcomponent*; ENPP2,* ectonucleotide pyrophosphatase/phosphodiesterase family member 2*; FN1,* fibronectin*; HGFA,* hepatocyte growth factor activator*; HPX,* hemopexin*; HRG,* histidine-rich glycoprotein*; H2BC12L,* Histone H2B type F-S*; H2BC14,* histone H2B type 1-M*; IGFBP4,* insulin-like growth factor-binding protein 4*; IGHV1OR15-1,* immunoglobulin heavy variable 1/OR15-1 (non-functional) (Fragment)*; IGHV6-1,* immunoglobulin heavy variable 6–1*; IGLV3-21,* immunoglobulin lambda variable 3–21*; IGKV1-9,* immunoglobulin kappa variable 1–9*; ITIH2,* inter-alpha-trypsin inhibitor heavy chain H2*; ITIH3,* inter-alpha-trypsin inhibitor heavy chain H3*; ITIH4,* inter-alpha-trypsin inhibitor heavy chain H4*; MASP2,* mannan-binding lectin serine protease 2*; PAPPA2,* pappalysin-2*; PDHX,* pyruvate dehydrogenase protein X component mitochondrial*; SERPINA5,* plasma serine protease inhibitor*; SVEP1,* sushi von Willebrand factor type A EGF and pentraxin domain-containing protein 1*; TF,* serotransferrin*; TMSB4X,* thymosin beta-4*; VWF,* von Willebrand factor.

Overall, the differences in proteome profiles between EOPE or LOPE and healthy pregnancy were largely associated with impaired angiogenesis and included perturbed abundance of inter-alpha-trypsin inhibitor heavy chain H2-4 (ITIH3 in EOPE and ITIH2/4 in LOPE)^8^, insulin-like growth factor-binding protein 4 (IGFBP4)^9–12^ and histidine-rich glycoprotein (HRG)^13–15^ (Fig. 2a). Immunoglobulin heavy variable 1/OR15-1 (IGHV1OR15-1) was the only protein differentially abundant between the two subtypes of preeclampsia, being substantially higher in abundance in EOPE compared to LOPE (FC = 14.29, FDR = 0.005) (Fig. 2b). Through our targeted proteomics we validated the changes observed with ITIH3 in EOPE (logFC = 0.429, *p* = 0.02) and ITIH 2&4 in LOPE (logFC = 0.290, *p* = 0.005 and logFC = 0.3498, *p* = 0.005, respectively). ITIH4 was also significantly upregulated in EOPE (logFC = 0.294, *p* = 0.02) therefore making ITIH2 uniquely perturbed in LOPE (Table 3). Interestingly, between EOPE and LOPE, IGHV1OR15-1 was not significantly different in abundance using the targeted proteomics but rather APOC3 (logFC = − 0.939, *p* < 0.0001) and HRG (logFC = 0.671, *p* < 0.0001). HRG is a well-studied biomarker of preeclampsia related to platelet haemostasis ^16–18^ that was increased ~ twofold in both phenotypes of preeclampsia (EOPE: FDR = 0.008; LOPE: FDR = 0.004) when we applied untargeted proteomics. However, following validation, HRG showed similar trend in EOPE (*p* = 0.007) versus healthy controls whereas it was downregulated in LOPE (*p* = 0.005) versus healthy controls and as such it was differentially abundant between the two phenotypes.

Furthermore, there were 11 DE proteins commonly shared between EOPE and LOPE. Immunoglobulin lambda variable 3–21 (IGLV3-21) was the top differentially abundant protein shared between EOPE (FDR = 0.002) and LOPE (FDR = 0.004), with an approximately 50% decrease in its abundance in either of phenotypes compared to healthy pregnancy controls. This was also confirmed as part of validation using targeted proteomics. Well-characterised preeclampsia biomarkers including fibronectin 1 (FN1)^19,20^ and complement factor D (CFD)^21,22^ were ~ twofold increased in abundance and among the most significant abundant proteins in both EOPE and LOPE, compared to control. Whilst CFD did not undergo validation, using targeted proteomics FN1 was only significant in EOPE (*p* = 0.007) and not in LOPE, versus healthy controls.

The proteins displaying significant changes of abundance were highlighted in a three-dimensional plot (Fig. 2b; Supplementary data 3). Among the proteins with significant fold changes in EOPE compared to healthy controls, a series of proteins have previously been reported as biomarkers of preeclampsia, including serpin family A member 5 (SERPINA5)^23^, pappalysin 2 (PAPPA2)^24,25^, hepatocyte growth factor activator (HGFAC)^26,27^ and thymosin beta-4 (TMSB4X)^28^. Whilst SERPINA5, PAPPA2, and TMSB4X did not undergo validation using targeted proteomics, HGFAC was not statistically significant as part of the validation (Table 3). IGFBP4, a protein significantly increased in abundance in EOPE (log_2_FC = 4.25, FDR = 0.0003) and LOPE (log_2_FC = 4.91, FDR = 0.004), compared to controls, is known to have anti-angiogenic properties^9–12^, adding further evidence to the central role of impaired angiogenesis in preeclampsia.

### Pathogenic pathway associated with different phenotypes of preeclampsia

**Figure 3:**
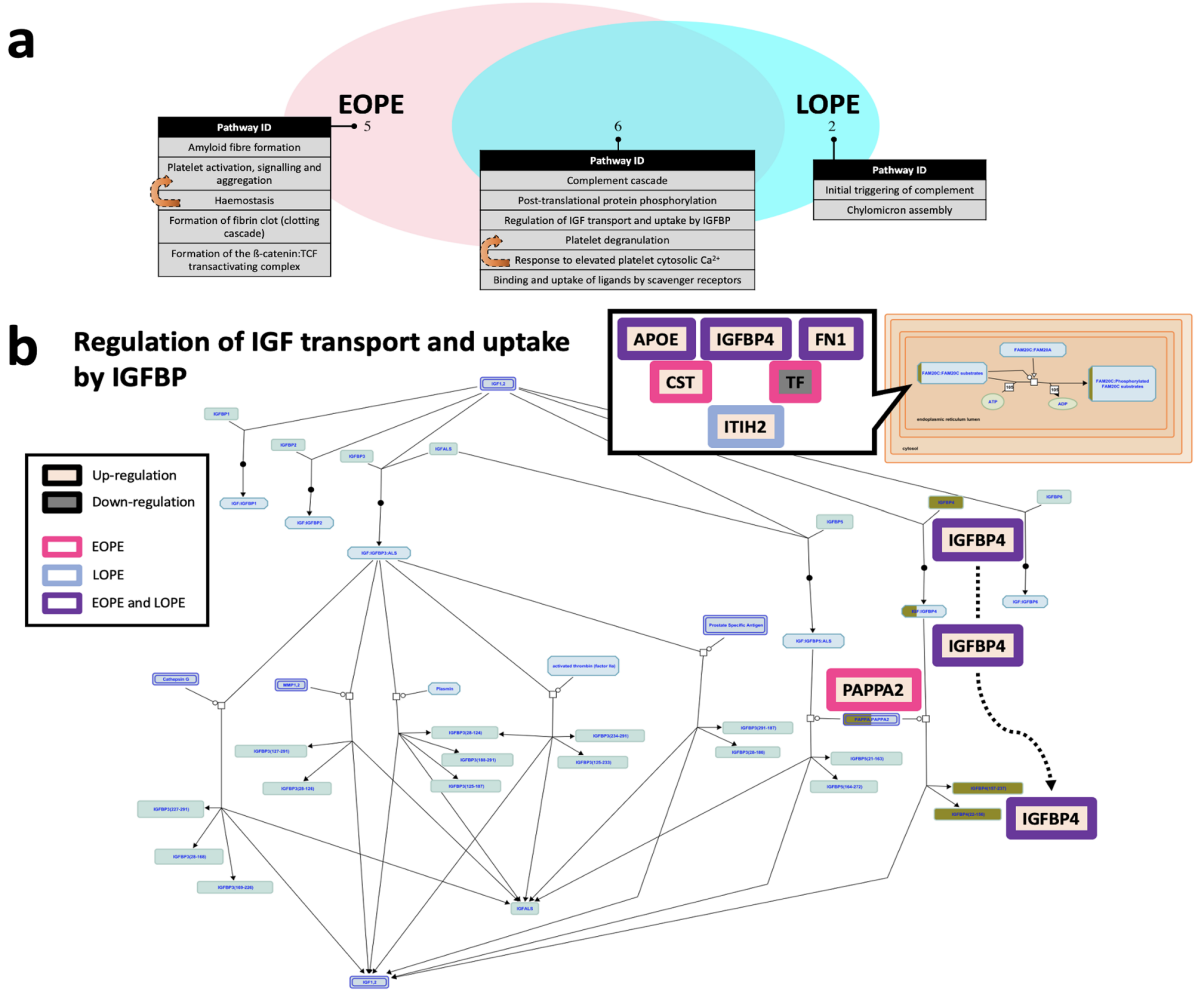
Pathway analysis. (**a**) Triple Venn diagram illustrating the number of pathways significantly changed in EOPE versus healthy pregnancy and LOPE versus healthy pregnancy. (**b**) Illustrative example of DE protein perturbations in the regulation of IGF transport and uptake through IGFBP. *IGF* insulin-like growth factor, *IGFBP* insulin-like growth factor binding protein. Source data are provided as a Source Data file.

Following identification of differentially abundant biomarkers for different phenotypes of preeclampsia, pathway enrichment analysis was performed for EOPE or LOPE group proteins, compared to healthy controls. Pathways altered in EOPE and LOPE (FDR < 0.05) were presented using a triple Venn diagram (Fig. 3a; Supplementary data 4). The pathway Venn diagram revealed a range of pathways associated with altered haemostasis and immune system.

A total of 13 pathways were significantly enriched, with 6 pathways shared between EOPE and LOPE groups (Fig. 3a), compared to healthy controls. Differentially abundant proteins are part of the biological pathway regulating insulin-like growth factor (IGF) transport and uptake through IGF binding protein (IGFBP; Fig. 3b), which plays a significant role in both EOPE (FDR = 0.01) and LOPE (FDR = 0.002). Given the pro-angiogenic function of IGF signalling pathway^29,30^, our findings emphasised the importance of impaired angiogenesis in the pathophysiology of preeclampsia. In addition, differentially abundant proteins were part of a series of haemostatic pathways, particularly platelet degranulation in response to elevated intra-platelet Ca^2+^ pathway, which were more pronounced in EOPE (FDR = 0.00001; Supplementary data 4). Proteins shown as differentially abundant in EOPE compared to healthy controls were more closely associated with acute inflammatory pathways than those identified in LOPE.

### Signalling networks in EOPE and LOPE

**Figure 4:**
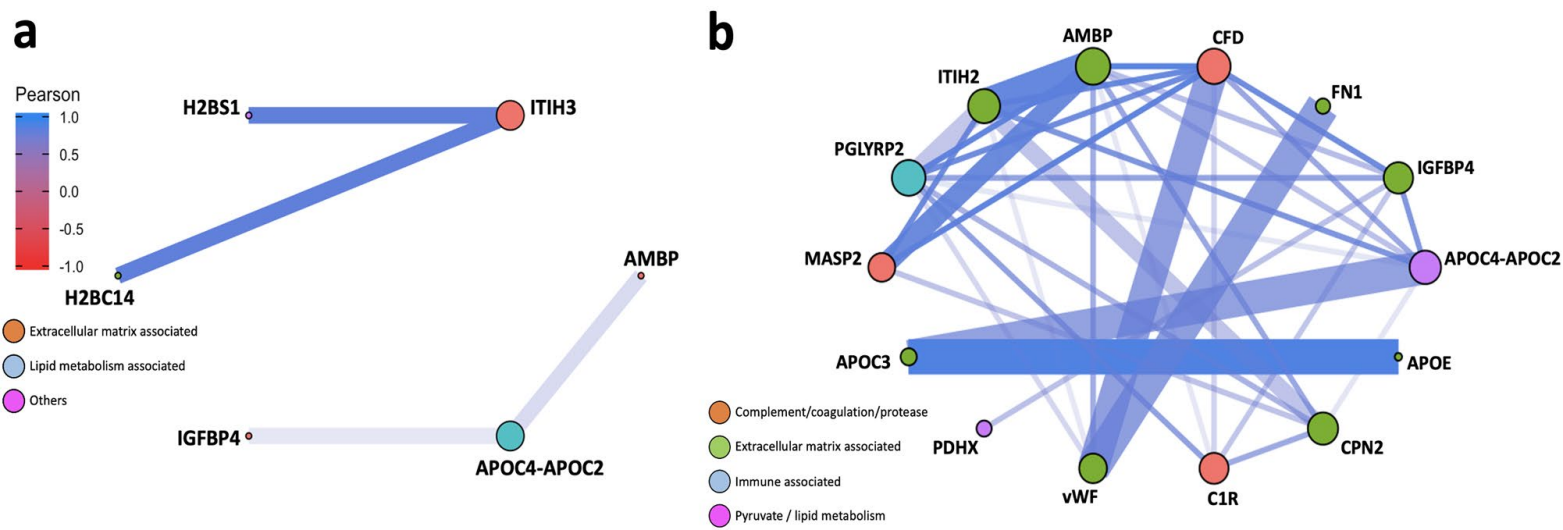
Network analysis. Network plots illustrating protein–protein interactions in (**a**) EOPE versus healthy pregnancy and (**b**) LOPE versus healthy pregnancy. Each node represents one protein; and each edge represents a pairwise Pearson correlation between proteins. The networks only show Pearson correlation coefficient > 0.7 or < − 0.7. Node: colour coded according to the functional classification of proteins annotated by DAVID database; size proportional to the corresponding eigenvector centrality (network influence). Edge: colour and length proportional to the Pearson correlation coefficient; width proportional to the scores derived from STRING database. Source data are provided as a Source Data file.

Pairwise correlation network analysis was next performed to investigate protein–protein interactions (PPIs) in EOPE and LOPE. Networks were highlighted with the most correlated nodes (Pearson correlation *r* > 0.7 or < − 0.7), where the colour and the length of the edge are proportional to the Pearson correlation coefficient *r* (Fig. 4a,b). To compare the PPIs in our data with broad reference evidence, the edge width was coded proportionally to the PPI-confidence scores derived from the STRING database^31^. Therefore, if consistent with the references, a pair of nodes are presented with a thick and opaque edge in between, or vice versa. Overall, our findings in terms of PPIs were consistent with those reported by published literature (Fig. 4a,b; Supplementary data 5).

To aid the readability of networks, the size of each node was programmed proportional to the corresponding eigencentrality (the influence in the entire network). Additionally, we also annotated the functional classification of each protein using DAVID^32,33^, as indicated by the node colours.

As shown in the networks, all pairwise correlations were positive. The PPI network in EOPE is simple, suggesting more direct pathogenesis in EOPE than LOPE (Fig. 4a,b). Proteins associated with extracellular matrix (ECM) were the key abundant proteins in EOPE, and lipid metabolism associated proteins appear to play a more important role in LOPE, although there was some overlap between the two phenotypes. ITIH3 and APOC4-APOC2 were identified as having a substantial influence within the PPI network in EOPE. LOPE was more closely associated with a range of complement/coagulation, ECM, immune associated and lipid metabolism proteins including APOC4-APOC2, CPN2, IGFBP4 and ITIH2. As part of the targeted proteomics validation, out of a total of 17 detectable proteins, in LOPE, the following proteins remained significantly different compared to control: ITIH2, APOC3, APOE, APOC4-APOC2, CPN2, C1R. On the other hand, FN1, MASP2 and vWF were no longer significantly abundant in LOPE versus healthy controls. APO-related proteins showed a strong correlation with previous studies depicted by the line thickness. In EOPE versus healthy control validation analysis, ITIH3 was significantly different whereas no significant difference was observed with APOC4-APOC2.

## Discussion

Systemic angiogenic imbalance in the maternal circulation likely linked to placental dysfunction is one of the most well-characterised pathogenic mechanisms in preeclampsia^34,35^. Although pregnancy and post-pregnancy complications and morbidities associated with the two phenotypes of preeclampsia are substantially different, the molecular insights underpinning these differences are currently lacking. Using plasma samples from age- and BMI-matched patients with EOPE or LOPE and normotensive women with healthy pregnancy, we identified a core set of plasma biomarkers and signalling pathways in EOPE and LOPE, compared to controls and adjusted for differences in gestational age. Published literature based on two independent longitudinal studies have already reported on the predictive markers for EOPE or LOPE based on proteomics analyses^36,37^. In this study, we used samples collected post-diagnosis of preeclampsia to better understand the pathophysiology after preeclampsia develops related to specific phenotypes that could identify potential therapeutic targets and inform future development of personalised treatments. This is very important given there is currently no cure for this condition.

For this study, we chose not to employ any depletion methods to remove highly abundant plasma proteins to prevent co-depleting any differentially abundant proteins, an approach that is becoming the standard in plasma/serum analysis^38^. In light of initially employing an unbiased untargeted proteomics approach, the currently utilised biomarkers, soluble fms-like tyrosine kinase-1 (sFlt1) and placental growth factor (PIGF)^39^ were not directly detected in this study, instead correlated proteins such as vascular endothelial growth factor receptor 3 (Flt4) were present. Follow-up studies will employ the more increasingly employed data-independent acquisition (DIA) with extensively fractionated pooled samples as a reference library to increase the depth of proteome analysis while managing instrument time. Nevertheless, we conducted targeted proteomics using similar samples to validate seventeen proteins of interest. The vast majority of the proteins showed the same pattern of abundance with only a few proteins showing some differences between targeted and untargeted proteomics analyses.

As mentioned above, the systemic biomarkers commonly utilised clinically for short-term prediction and diagnosis of preeclampsia include the markers of angiogenesis, sFlt1 and PIGF^39^. In line with the importance of impaired angiogenesis in the pathogenesis of preeclampsia, we discovered a series of angiogenic proteins, differentially regulated by EOPE or LOPE including: (i) ITIH2^8^ only perturbed in LOPE compared to control; (ii) ITIH3&4 and (iii) IGFBP4^9–12^, all perturbed in EOPE or LOPE, compared to controls. Interestingly, in the discovery/untargeted proteomics sample set, HRG was upregulated in both EOPE and LOPE, whereas in the validation/targeted proteomics sample set, HRG was upregulated in EOPE and downregulated in LOPE, compared to controls. HRG was also differentially abundant between EOPE versus LOPE. This was unexpected however it could be due to factors such as differences in the peptide selection in targeted vesus untargeted proteomics. Aligned to our findings, HRG was previously shown to be significantly higher in placental tissues from women following diagnosis of EOPE^20^, whereas there appear to be no available reports looking specifically at its regulation in LOPE. On the other hand, early in pregnancy in women who later proceeded to develop preeclampsia (EOPE and LOPE grouped together), HRG was lower than in normal pregnancies^18^. The biomarker and therapeutic target potential of HRG should be confirmed in future studies in both EOPE and LOPE. Differences in angiogenic profile between EOPE and LOPE are consistent with Schaarschmidt et al.’s study^40^, where the progressive increase of sFlt1 and sFlt1/PIGF ratio was more prominent in EOPE than LOPE. Therefore, it is likely that higher levels of these biomarkers are more representative of EOPE, which should be explored in the future. Other angiogenesis-related biomarkers recently identified, FKBPL and CD44, appear to be associated with both types of preeclampsia^41^. Our study is one of the first studies to provide molecular insights at the proteome level related to angiogenic pattern between EOPE and LOPE, and healthy controls in the same study.

Our proteomics analysis identified the angiogenic IGF signalling pathway as perturbed in both EOPE and LOPE, compared to healthy controls. There is strong evidence supporting the role of this regulatory pathway in maternal circulation in preeclampsia^42^. Dysregulation of the IGF pathway plays a role in inadequate trophoblast invasion of the maternal decidua, resulting in inappropriate placentation^43^. Considering the importance of the angiogenic balance, our findings provide a mechanistic insight into the complex interplay between pro- and anti-angiogenic factors of IGF signalling pathway in preeclampsia. IGFBP4, one of the core components of the IGF signalling pathway, was substantially increased in abundance in both EOPE and LOPE. The elevation of IGFBP4 levels is reflective of restrictive angiogenesis^10,11^. PAPPA2, a protease of IGFBP5^42^, was also highly overexpressed in EOPE compared to control. Although a number of studies reported up-regulation of PAPPA2 in maternal circulation in preeclampsia^44–46^, we demonstrated the specific increase of PAPPA2 levels in EOPE, compared to healthy controls. We did not identify IGFBP5 in our plasma proteomics analysis, which could be due to the excessive cleavage by overexpressed PAPPA2. Likewise, the increase of PAPPA2 levels in maternal circulation may compensate for the suppression of IGF signalling pathway due to up-regulated IGFBPs, which was also suggested by Nishizawa et al.’s study^46^. Whilst IGFBP4 and PAPPA2 were not on the list of validated proteins, the results are convincing given the plethora of data already available related to these proteins in preeclampsia, which are also aligned to our findings.

Using untargeted proteomics, APOE, APOC4-APOC2 and APOC3 were all significantly increased in abundance in LOPE whereas APOE and APOC4-APOC2 were borderline increased in EOPE (*p* = 0.046). Interestingly, following validation, none of these were significantly abundant in EOPE in contrast to LOPE. Therefore, it is clear that APO-associated proteins are more relevant to the pathogenesis of LOPE. In fact, targeted proteomics showed that APOC3 was differentially abundant between EOPE and LOPE with its abundance being reduced in EOPE compared to LOPE. The importance of these findings should be explored in future studies by specifically looking at the differences between these two phenotypes of preeclampsia.

APOE is well-known for its protective role in atherosclerosis, and APOE-knockout (KO) mouse model is often used as pre-clinical atherosclerosis model^47^. Interestingly, multiple in vivo studies suggested that IGFBP4 could also have a protective role in atherosclerosis^48–52^. For example, when PAPPA, the protease of IGFBP4, was genetically overexpressed in atherosclerosis models (APOE-KO), IGF bioavailability was enhanced due to reduced IGFBP4, resulting in a more severe atherosclerotic plaque formation^51^. In preeclampsia, lipid deposition in spiral uterine arteries resembles the early stage of atherosclerosis^53^. Our findings show that the pathways associated with atherosclerosis were present in both EOPE and LOPE, however in EOPE particularly pronounced pathways are related to platelet activation, signalling and aggregation, whereas in LOPE the signalling pathways are more aligned to lipoprotein/chylomicron assembly, transport and metabolism. The different extents of atherosclerotic pattern may explain the differences in the symptoms and the risk of future cardiovascular events between EOPE and LOPE^54^.

In this study, complement and immune system impairment was implicated in both LOPE and EOPE. However, we discovered two novel ITIH protein biomarkers of preeclampsia, which have not been reported previously; ITIH3 and ITIH4 were both increased in EOPE and LOPE whereas ITIH2 was the only one increased in LOPE. Exacerbated maternal systemic inflammation is one of the key factors in the pathophysiology of both phenotypes of preeclampsia^55–57^. ITIH proteins play a contradictory role in inflammation, acting as both pro- and anti-inflammatory acute-phase protein^56^. The most well-known function of ITIH proteins is linked to their capability to inhibit tumour growth and metastasis, which is largely dependent on their ability to stabilise ECM and their covalent binding to hyaluronic acid (HA), a pro-angiogenic ligand when degraded^58,59^. The elevation of ITIH proteins may indicate an inhibitory effect on degradation of ECM and the HA-CD44 pathway, which in turn leads to anti-angiogenic effect^58,59^. Recently, a study demonstrated predictive and diagnostic biomarker potential of a novel anti-angiogenic FKBPL pathway via CD44, in preeclampsia^41^. Specific elevation of ITIH3/ITIH4 and ITIH2 in both preeclampsia phenotypes and LOPE, compared to healthy controls, respectively, likely reflects molecular differences related to the pathogenesis of these different phenotypes of preeclampsia that could be explored in the future and as specific systemic biomarkers of EOPE or LOPE.

Although our study is only of the few that sheds light on important pathophysiological differences between two different subtypes of preeclampsia, there are some limitations. Validation samples were not from an entirely independent cohort, therefore the biomarkers and targets we identified should be confirmed in the future even though there was a strong alignment with the published literature. Although a number of proteins were confirmed through targeted proteomics, there were some differences between untargeted and targeted proteomics results particularly when comparing EOPE and LOPE directly, in terms of HRG and APOC3 and IGHV1OR15-1. These biomarkers should be further validated in an independent cohort of EOPE and LOPE using plasma samples. Also, there was a difference in gestational age between the preeclampsia and control groups and even though we accounted for this statistically, this is something that could have influenced the results.

In conclusion, the plasma proteome profiles of preeclampsia revealed changes in the abundance of a series of angiogenesis, inflammation/immune, lipid metabolism and coagulation pathways. The IGF signalling pathway appears to play a key role in regulating the systemic angiogenic imbalance in preeclampsia. Although there is substantial overlap of the pathogenic pathways between EOPE and LOPE, the mechanisms appear different. We also report on an ITIH2 protein and APO-related proteins, specific for LOPE that could be explored in the future for personalised management of preeclampsia. Overall, our untargeted and targeted proteomics analyses of the plasma proteome identified a number of novel biomarkers and pathogenic pathways in preeclampsia, specifically emphasising differences between EOPE and LOPE and the importance of perturbed angiogenesis, lipid metabolism/transport, vascular homeostasis and inflammation in this condition.

## Materials and methods

### Patient recruitment and sample collection

Blood samples were collected from healthy normotensive pregnant women (n = 18) and age and body mass index (BMI)-matched women diagnosed with EOPE (n = 17—untargeted proteomics; n = 14—targeted proteomics) or LOPE (n = 11—untargeted proteomics; n = 14—targeted proteomics). Diagnosis of preeclampsia was carried out according to the 2013 ACOG guidelines^1^. EOPE was diagnosed before 34 weeks of gestation whereas LOPE was diagnosed from 34 weeks of gestation. Blood sampling took place upon hospital admission for delivery. Blood was collected in EDTA coated tubes. Following collection, the samples were centrifuged at 3000g for 10 min to collect plasma. Pregnant women with multiple pregnancies, pre-existing hypertension, diabetes mellitus, cardiovascular and renal conditions complicated with proteinuria, were excluded from the study.

All participants provided written informed consent prior to inclusion in the study. The study was approved by the local institutional human ethics review boards including the University of Technology Sydney, the University of Nis and all the local hospital human ethics committees in accordance with the Declaration of Helsinki and the National Statement on Ethical Conduct in Human Research (Australia).

### Proteomics

**Figure 5:**
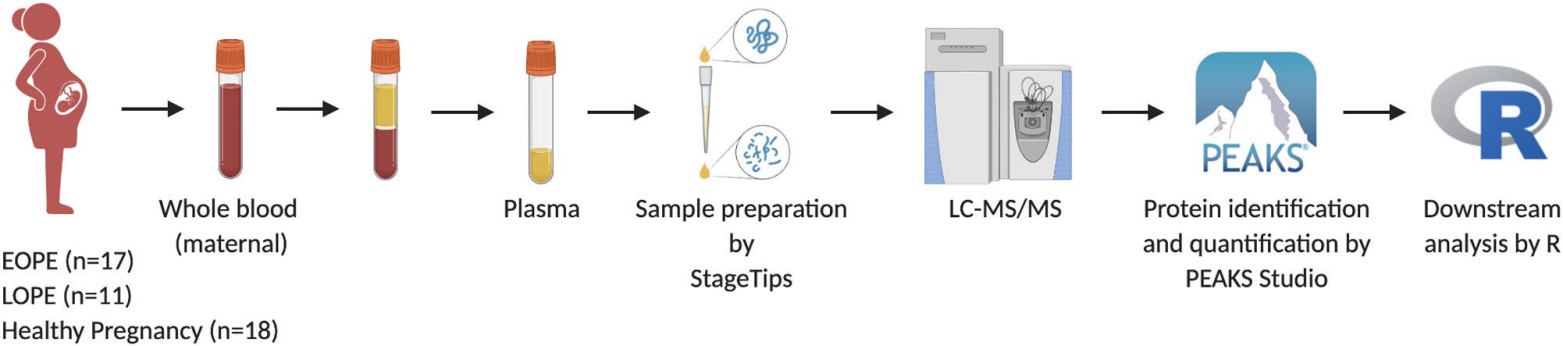
Overview of the experimental design and data analysis.

Plasma sample preparation was adjusted from the previously described methods using STop-And-Go-Extraction tips (STAGE Tips)^60,61^. The overview of the study and experimental design as well as data analysis is depicted in Fig. 5. Briefly, 1 μL (~ 50–60 μg of protein determined by BCA assay) of plasma was diluted in lysis buffer composed of 50 mM Tris–HCl pH 8.8, 1% sodium deoxycholate, 5 mM Tris (2-carboxyethyl) phosphine hydrochloride (reducing agent) and 20 mM acrylamide monomers (alkylation agent), at a sample to lysis buffer ratio of 1:25, and heated to 95 °C for 10 min for denaturation, reduction and alkylation. The lysates were then diluted 1:9 with water. Proteins were digested in sequencing grade trypsin (1 μg/μL; Promega, USA) overnight at 37 °C, at an enzyme to substrate ratio of 1:50. The digestion was ceased by equal volume of ethyl acetate containing 1% trifluoroacetic acid (TFA).

The peptide mixtures were then cleaned, concentrated and enriched through STAGE Tip. The STAGE Tip was made by a core extracted from a 47 mm styrene divinyl benzene-reversed phase sulfonate (SDB-RPS Empore, 3 M) disk using a 14G flat-ended needle. The core was then inserted into an ordinary 100 μL pipette tip. Prior to sample addition, the tips were activated with 100 μL 100% acetonitrile and centrifuged at 1000 × g for 1 min. The tips were equilibrated with 100 μL 0.1% TFA in water and 30% methanol/1% TFA and centrifuged at 1000 × g for 3 min. The two phases of each sample were mixed by vigorous vortex, followed by removal of the volume equivalent of ~ 10 μg digested peptides which was loaded onto a STAGE Tip. The loaded peptides were washed twice by 100 μL 99% ethyl acetate/1% TFA, followed by a wash with 100 μL 0.2% TFA in water. To elute, 100 μL 5% ammonium hydroxide/80% acetonitrile was added to each tip and centrifuged at 1000 × g for 5 min into an autosampler vial insert (Thermo Fisher, USA). Samples in the vial inserts were dried in SpeedVac vacuum concentrator (Thermo Fisher) at highpower for 1 h. The peptides were then resuspended in 25 μL 0.1% formic acid to a concentration of 0.4 μg/μL.

The setup of mass spectrometry was previously described^62^. Using an Acquity M-class nanoLC system (Waters, USA), 5 µL of the sample was loaded at 15 µL/min for 3 min onto a nanoEase Symmetry C18 trapping column (180 µm × 20 mm) before being washed onto a column with a laser pulled emitter (75 µmID × 350 mm) packed with SP-120–1.7-ODS-BIO resin (1.7 µm, Osaka Soda Co, Japan) heated to 45 °C. Peptides were eluted from the column and into the source of an Q Exactive™ Plus mass spectrometer (Thermo Fischer) using the following program: 5–30% MS buffer B (98% acetonitrile + 0.2% formic acid) over 90 min, 30–80% MS buffer B over 3 min, 80% MS buffer B for 2 min, 80–5% for 3 min. The eluting peptides were ionised at 2400 V. For discovery proteomics, a Data Dependant MS/MS (dd-MS^2^) experiment was performed, with a survey scan of 350–1500 m/z performed at 70,000 resolution for peptides of charge state 2^+^ or higher with an AGC target of 3e6 and maximum injection time of 50 ms. The top 12 peptides were selected fragmented in the HCD cell using an isolation window of 1.4 m/z, an AGC target of 1e5 and maximum injection time of 100 ms. Fragments were scanned in the Orbitrap analyser at 17,500 resolution and the product ion fragment masses measured over a mass range of 120–2000 m/z. The mass of precursor peptides was then excluded for 30 s. For targeted validation, a Parallel Reaction Monitoring (PRM) experiment was performed with a survey scan of 350–1500 m/z performed at 70,000 resolution for peptides of charge state 2^+^ or higher with an AGC target of 3e6 and maximum injection time of 50 ms before acquisition of m/z of the target ions, which were scheduled in a 10 min window around the retention time determined in the discovery experiment. Each m/z value in the target inclusion list (Supplementary tables 6,7,8) was selected fragmented in the HCD cell using an isolation window of 1.4 m/z, an AGC target of 2e5, a maximum injection time of 50 ms, normalised collision energy of 27 and a loop count of 20 before performing another survey scan. Fragments were scanned in the Orbitrap analyser at 17,500 resolution and the product ion fragment masses measured over a mass range of 120–2000 m/z.

The MS/MS data files for the discovery proteomics experiment were searched using PEAKS Studio X + (Bioinformatics Solutions, CA) against the human proteome database (2019) and a database of common contaminants with the following parameter settings: fixed modifications = none; variable modifications = propionamide (cysteine modified with acrylamide monomer), oxidised methionine, deamidated asparagine; enzyme = semi-trypsin; number of allowed missed cleavages = 3; peptide mass tolerance = 10 ppm; MS/MS mass tolerance = 0.05 Da. The results of the search were then filtered to include peptides with a − log_10_*P* score that was determined by the False Discovery Rate (FDR) of < 1%, in which decoy database search matches were < 1% of the total matches. The data files for the PRM experiment were analysed using Skyline (version 21.2.0.536) using a spectral library created from a pooled sample of all analysed samples.

### Statistics

All statistical analyses were performed in R (4.0.2). All proteomic data were total ion count (TIC)-normalised, followed by log_2_-transformed prior to entering pipelines in R. The packages or functions utilised in this study included: (i) limma package^63^ for DE analysis of comparisons between EOPE, LOPE and healthy control groups, while adjusting for gestational age (GA) at delivery, (ii) geneSetTest function in limma package for pathway analyses annotated by Reactome database^64^, and (iii) igraph package for network analyses of protein–protein interactions (PPIs) annotated by pairwise Pearson correlation, PPI scores in STRING database^31^ and protein functions in DAVID database^32,33^. Relative quantification of proteins or pathways between subtypes was expressed as fold change (FC). The Benjamini–Hochberg method was used to calculate the adjusted *P* values, also known as FDR, for all aforementioned analyses. DE proteins were defined as FDR < 0.05.

### Ethics approval and consent to participate

All participants provided written informed consents prior to inclusion in the study. The study was approved by the local institutional human ethics review boards in accordance with the Declaration of Helsinki and the National Statement on Ethical Conduct in Human Research (Australia). All other authors have nothing to disclose.

## Supplementary Information

Supplementary Information 1.

Supplementary Information 2.

Supplementary Information 3.

Supplementary Information 4.

Supplementary Information 5.

Supplementary Information 6.

Supplementary Information 7.

Supplementary Information 8.

## Data Availability

The mass spectrometry proteomics data have been deposited to the ProteomeXchange Consortium with the dataset identifier PXD026931. Also, the authors provided a publicly available, interactive online application of supporting data for easy navigation (https://hao-chen-uts-99171821.shinyapps.io/PEplasmaproteomics/_w_1022d2ef/). Username and password can be obtained from the corresponding author upon reasonable request.
